# Hereditary risk factors for the development of gastric cancer in younger patients

**DOI:** 10.1186/1471-230X-4-28

**Published:** 2004-10-27

**Authors:** Mohammad Yaghoobi, Naser Rakhshani, Farhad Sadr, Raheleh Bijarchi, Yasamin Joshaghani, Ashraf Mohammadkhani, Arezou Attari, Mohammad Reza Akbari, Mahshid Hormazdi, Reza Malekzadeh

**Affiliations:** 1 Digestive Disease Research Center, Tehran University of Medical Sciences, Shariati Hospital, Kargar Shomali St, Tehran 14114, Iran

## Abstract

**Background:**

It is believed that the development of gastric cancer (GC) before the age of 50 has a hereditary basis. Blood group A and history of gastric cancer in first-degree relatives have been shown to be risk factors for GC.

**Methods:**

In this case-control study, we enrolled patients with GC who were diagnosed before the age of 50. Patients who were diagnosed as having GC were selected. A total of 534 cases were found; of these, 44 diagnosed before the age of 50 were included in the case group. For the control group, 22 males and 22 females were randomly selected from the remaining subjects, who had diagnoses of GC after the age of 50. All the surviving patients and family members of the dead patients were interviewed about the history of cancer in the family and the age at which other family members developed cancer. The blood group of each subject was also obtained.

**Results:**

forty-four cases under 50 years old (mean age: 36.2 years) and forty-four controls (mean age: 67.1 years) were enrolled in the study. At the time of the study, 59.1% of the study group and 50% of the control group were alive (P value = NS). In the study group, 68.1%, 13.6%, 13.6% and 4.5% had blood groups O, A, B and AB, respectively. In the control group the corresponding figures were 27.7%, 63.6%, 6.8% and 4.5%. First or second-degree relatives with cancer, including gastric (the most frequent), breast, lung, gynecological and hematological malignancies, were noted in 54.5% of the cases and 11.4% of the controls (p < 0.01). Family histories of cancer were accepted as valid provided that they were based on valid medical documents.

**Conclusions:**

It seems that the development of GC before the age of 50 is likely to be accompanied by familial susceptibility. Interestingly, our study showed a significant correlation between blood group O and the development of gastric cancer under the age of 50.

## Background

Gastric cancer is the second most common cause of cancer-related death in the world [1]. Its incidence varies considerably worldwide [2]. In general, it is a larger problem in developing countries than in industrialized nations, and shows a predilection for urban and lower socioeconomic groups [3,4]. The estimated crude rate accounts for approximately 9.9% of cancers worldwide [5]. Gastric cancer rarely occurs before the age of 40. The incidence rises steadily thereafter, peaking in the seventh decade. Men are nearly twice as susceptible as women. This cancer alone is the cause of more than 750,000 deaths per annum in the world [6]. Marked variation within countries has also been observed [3,4], particularly in high-risk countries [7]. In developing countries, the overall incidence of gastric cancer is increasing and projections indicate that the annual number of new cases will increase significantly during the next few decades as a result of adult population growth [6]. A recent cancer survey by the Iranian Ministry of Health and Medical Education revealed that gastric adenocarcinoma is the most common fatal cancer in Iran, with a wide variation of death rate among different provinces [8]. According to recent cancer statistics, deaths due to gastric cancer constitute about 39% of all deaths due to cancer each year in some parts of Iran [9].

The reduced incidence of gastric cancer in western countries reflects a decrease in cancers arising in the distal stomach (body and antrum). In contrast, the incidence of cancer in the proximal stomach and esophagogastric junction has steadily increased, at a rate exceeding that of any other cancer except melanoma and lung cancer [10-13]. In a very recent study, our group showed that cardiac cancer constitutes 49.5% of all sites for gastric cancer in Iran. In contrast, cancers of body and antrum comprise 20.6% and 29.9% respectively [9]. Unlike cancer of the distal stomach, cancers of the proximal stomach and esophagogastric junction are more common among higher socioeconomic classes [6]. Overall, these observations suggest that proximal cancers share a similar pathogenesis, which is distinct from that of distal cancers. Zanghieri and La Vecchia found that about 10% of cases show familial clustering. Epidemiological studies have shown that the risk of gastric cancer in first-degree relatives is increased 2- to 3-fold [14-17]. The relative contributions of inherited susceptibility and environmental effects on familial gastric cancer are poorly understood.

In general, familial genetic mechanisms do not play as important a role in gastric cancer as they do in e.g. colorectal cancer. Nonetheless, in some regions, a family history of gastric cancer may be a risk factor for the disease, although this might reflect environmental factors shared by members of a family [18]. Rate collections of familial aggregates of gastric cancer have been reported, but are distinctly unusual. As yet there is no comprehensive hypothesis for the development of gastric cancer. Gastric cancers are associated with chromosomal aberrations and other genetic defects, but none of these is necessary or sufficient for cancer to occur.

In a review about genetic predisposition to gastric cancer, Bevan and Houlston (1999) concluded that several genes may be associated with increased risk [19]. Gastric cancer is a manifestation of several inherited cancer predisposition syndromes including hereditary nonpolyposis colon cancer, familial adenomatous polyposis, Peutz-Jeghers syndrome and Cowden disease. This suggests the presence of predisposing genes with different effects.

Many studies have addressed the correlation between ABO antigens and the development of gastric cancer, but most of these have indicated a correlation between sporadic cases of gastric cancer and blood group A. This association further supports the role of genetic factors in the development of gastric cancer [21]. Blood type A is more strongly associated with the diffuse histopathological type of gastric cancer than the intestinal type [21,22]. To our knowledge, similar studies on the specific category of gastric cancer in younger patients are scanty. This may be one of the first studies on the role of hereditary factors in the development of the gastric cancer in younger patients.

## Methods

The study was designed as a case-control study. We set up an active surveillance to identify patients with gastric cancer. Patients' records in the department of pathology in the main private referral facility in Tehran were scrutinized for gastric cancer cases between 1999 and 2003. Patients are referred here from all regions of the country and from different ethnic backgrounds and they are operated upon in the same hospital, so all the operation and pathology reports were available simultaneously. All the pathology reports were prepared and diagnosed by the same pathologists. The cases were selected from patients who were diagnosed with gastric cancer before the age of fifty. The sex-matched controls were randomly selected and enrolled from patients who were diagnosed over the age of fifty. All the patients and their family were interviewed regarding the history of gastric or other types of cancer over three generations, and the blood groups of affected members were ascertained. Family histories of cancer were accepted as valid provided that they were based on valid medical documents. The transfusion records of the operation were also used to identify the patients' blood groups.

*Statistical analysis *was performed using the SPSS Statistical Package (version 10.0). The quantitative variables were expressed as means (minimum-maximum) when appropriate. A chi-square test was performed to ascertain the overall effect of blood group on the development of gastric cancer before the age of 50. All statistical tests were two-sided and differences at the 0.01 level were considered statistically significant.

## Results

**Figure 1:**
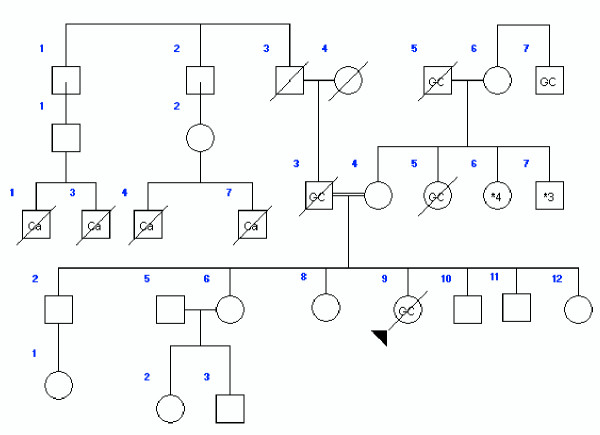
A family with aggregation with gastric cancer (GC: Gastric Cancer; Ca: History of gastric cancer but not confirmed by a pathologic reports for the histologic type of cancer).

**Figure 2:**
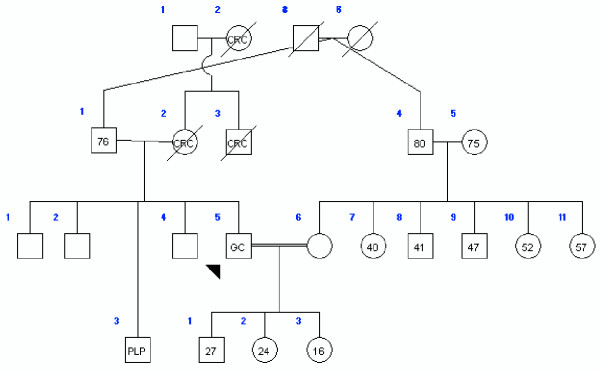
A family with a history of the aggregation with Colorectal cancer (GC: Gastric Cancer; CRC: Colorectal Cancer; PLP: Colorectal Polyps; Numbers in circles: Current age of the persons; CAG: Chronic Active Gastritis).

**Figure 3:**
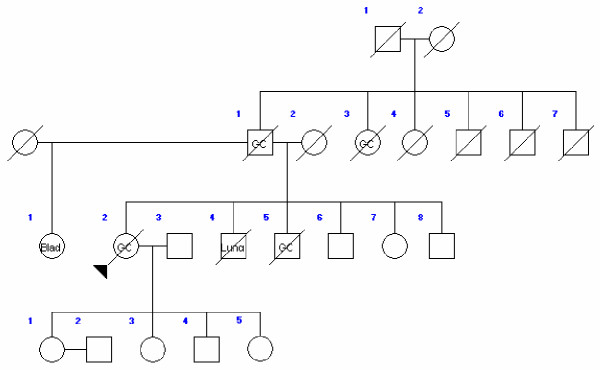
A family with a history of the aggregation with other cancers as well as gastric cancer (GC: Gastric Cancer; Lung: Lung Cancer; Blad: Bladder Carcinoma).

At the beginning of the study, 44 cases (mean age: 36.2, 18–49; m/f = 1) under 50 years old and 44 sex-matched controls (mean age: 67.1, 50–88) were enrolled. Table 1 shows the pathological characteristics of all 88 subjects. At the time of the study, 59.1% of the case group and 50% of the control group were alive; 53.8% of the case group and 38.6% were living in Tehran, but no information on residence background was available. Data regarding first-degree relatives were complete for both groups. These data comprised information on 383 persons in the case group (average 9.1 for each proband) and 498 in the control group (average 11.6 for each proband). Table 2 shows the distribution of blood groups in the two subject groups. Gastric (22 cases) and other types of cancer were reported in 54.5% of the first-degree relatives of the cases and 11.4% of the first-degree relatives of the controls (p < 0.01). The "other types of cancer" among relatives of the case group comprised colorectal (7 cases), breast (3 cases), lung (3 cases), gynecological (2 cases), hematological (1 case) and bladder (1 case) malignancies. For the control group, the corresponding figures were colorectal (2 cases), breast (1 case), lung (1 case) and prostate (1 case) malignancies. Figures 1, 2, 3 show three pedigrees of familial connections.

**Table 1:** The pathologic characteristics of the tumor in the case and control group (n = 44 in each group).

	Pathologic differentiation		Pathologic Type		Location of the tumor	
Case group	*Well differentiated*	1.4%	*Diffuse*	0.5%	*Cardia*	13.6%
	*Moderately differentiated*	5.0%			*Body*	18.2%
	*Poorly differentiated*	1.4%	*Intestinal*	7.3%	*Distal*	68.2%
Control group	*Well differentiated*	7.3%	*Diffuse*	5%	*Cardia*	22.7%
	*Moderately differentiated*	5.0%			*Body*	29.5%
	*Poorly differentiated*	7.7%	*Intestinal*	5%	*Distal*	47.7%
P value		NS		NS		NS

**Table 2:** Frequency of the different blood groups in the study population (*n *= *44*). *The figures in parentheses are the number of the patients*.

Blood group	O	A	B	AB
Cases	(30) 68.1%	(6) 13.6%	(6) 13.6%	(2) 4.5%
Controls	(11) 27.3%	(28) 63.6%	(3) 6.8%	(2) 4.5%
P value	<0.05	<0.01	NS	NS

## Discussion

This case control study demonstrates that hereditary factors, especially familial history of cancer and possession of blood group O, are associated with the development of gastric cancer under the age of fifty. To our knowledge, this may be the first study showing a correlation between blood group O and the development of gastric cancer in a specific category of patients.

Risk factors for gastric cancers have been explored in a number of previous studies, including genetic factors such as blood group. Haenszel et al. suggested an association between gastric cancer and blood type A, supporting the view that genetic factors have a role in the development of gastric cancer [21]. Our study emphasizes the role of genetic factors in one subcategory of patients, those who develop gastric cancer under the age of fifty. Blood group A is more strongly associated with the diffuse histopathological type of gastric cancer than the intestinal type [21,22]. In our study, most of the patients had a diffuse rather than intestinal type, so we could not test this association. A larger sample size would be needed. On the other hand, there might be a higher prevalence of Helicobacter pylori in our community, causing a higher incidence of the diffuse type of gastric cancer. However, there were no significant differences in histological type of gastric cancer between the case and control groups. In a study by Su et al. in 2001, a total of 6685 patients with esophageal carcinoma and 2955 patients with cardiac cancer in the Chaoshan district were retrospectively assessed for their association with ABO blood groups. Su et al. showed that the distribution of ABO blood groups in patients with esophageal carcinoma or cardiac cancer was similar to that in the normal local population, but there was an association between blood group B and the development of cancer of cardia in males [23].

In our study, approximately 54% of the case group had a familial history of cancer compared to 11% of the control group. This seems compatible with the findings of a population-based case-control study of stomach cancer in Warsaw, Poland. Here, the investigators interviewed 464 cases and 480 controls to evaluate the role of family history and other risk factors. A greater than threefold increase in risk was associated with a history of gastric cancer in a first degree relative (OR = 3.5), but no excess risk was seen for other forms of cancer. The risk associated with familial occurrence was not significantly modified by gender, age or ABO blood type, and did not vary with Lauren histological classification24. Despite the relatively large sample size in the Polish study, younger patients were not evaluated as a separate category. This may explain the difference between their results and ours. Furthermore, they defined "positive family history" as having a first-degree relative with gastric cancer. In contrast, we considered all types of malignancy in first and second-degree relatives; though the familial incidence of other (non-GI) malignancies in our study may reflect their higher frequency in the community rather than any genetic risk factor. The Polish study did not confirm previous results on the correlation between blood group A and gastric cancer. Moreover, another study by Parsonnet et al on 90 cases and 89 controls showed no association between ABO blood group and malignancy [25]. In a multicentric study in Italy, 1016 patients with gastric cancer and 1623 population controls were interviewed to determine family histories of gastric, esophageal and colorectal cancer. A significant association was found with history of gastric cancer in a sibling or parent (odds ratios 2.6 and 1.7, respectively). Among the adult siblings of controls and cases, the prevalences of gastric cancer reported at interview were 1 and 2.7%, respectively. A further increase was noted in families with at least one affected parent (1.4 and 5.7%). The risk of gastric cancer associated with a positive family history was greater (increased about 2-fold) among residents of low-risk areas. Among the cases, there was no relationship between family history of gastric cancer and blood group A or histological type according to the Lauren classification [26]. In our study, there was no significant relationship between the histological type of the cancer and positive family history or blood group. However, this does not prove that the two variables do not correlate; an association might become apparent with a larger study. Mecklin et al studied the clinical and histopathological characteristics of gastric carcinoma in young patients (under 40 years old) in Finland in 1988. In 94% of the young patients, the carcinoma was of the diffuse type. They showed a poor prognosis, an equal sex ratio, and a strong association with blood group A in their study group. They also found a highly significant over-representation of gastric cancer in the parents of the index cases (p < 0.001) [27]. The difference between Mecklin's study and ours in the blood groups identified as risk factors may reflect ethnic differences; both studies confirm a significant correlation between a specific blood group and the development of gastric cancer. Future studies may use linkage analysis to detect genetic abnormalities in chromosomal regions that are located near the genes encoding the ABO antigens.

Matching the geographical origins of the cases and controls could have improved the power of our study by excluding ethnic factors from the study population. However, this is very difficult to achieve in such studies because there is a high rate of combinations between races in the country. In addition, the sample size was too small for such effects to be excluded. However, there were no significance differences between the two groups in respect of the origins of the subjects.

In conclusion, our results show that familial history of cancer, and hereditary factors including blood group, have a role in the development of gastric cancer in young patients. The role of environmental factors may be more important in older patients and can be considered in future studies.

## Competing interests

The authors declare that they have no competing interest.

## Authors' contributions

MY designed and assisted in the conduction of the study, analyzing of the data and draft the manuscript.

NR, FS, RB and YJ assisted in the conducting and designing the study and interview with the study cases as well as drafting manuscript.

AM and MA assisted in analyzing of the study and conducting interviews.

AA and MH reviewed and approved the pathology reports of the patients and case collecting.

RM supervised the study scientifically and executively and assisted in drafting the manuscript.

All authors read and approved the final manuscript.

## Pre-publication history

The pre-publication history for this paper can be accessed here:


